# Non-compliance of Spectacle Wear in School-Going Children With Refractive Errors

**DOI:** 10.7759/cureus.52702

**Published:** 2024-01-22

**Authors:** Muhammad Irtza, Rabiah Mahwish, Muhammad Mohamin, Muhammad Shaheer Afzal, Muhammad Qasim Ali, Muhammad Shaheer Waqar

**Affiliations:** 1 Medical School, Services Institute of Medical Sciences Lahore, Lahore, PAK; 2 Community Medicine, Khawaja Muhammad Safdar Medical College, Sialkot, Sialkot, PAK

**Keywords:** world health organization (who), glasses, pakistan, lahore, children, spectacle wear difficulties, refractive errors, school-going children, non-compliance, spectacle wear

## Abstract

Background

Uncorrected refractive errors are the most common cause of avoidable visual impairment in children worldwide. The school screening of refractive errors is one of the most important initiatives outlined in WHO Vision 2020 targets for control of avoidable visual impairment in children. However, the benefit depends on the compliance of the spectacle worn by children.

Objective

To determine non-compliance of spectacle wear and its predisposing factors among school-going children in Lahore, Pakistan.

Methods

This cross-sectional analytical study was conducted among 200 school-going children (5-16 years), with spectacle prescription for at least the last six months studying in primary and secondary schools of Lahore, by using convenience sampling. We collected data with the help of a standardized, self-administered, close-ended questionnaire determining age, gender, class, and non-compliance and its reasons. Data were subjected to statistical evaluation using Statistical Product and Service Solutions (SPSS, version 26; IBM SPSS Statistics for Windows, Armonk, NY), and a chi-square test was applied to determine the statistical significance. p-value 0.05 was considered significant.

Results

Of the 200 children, 42 were boys, and 158 were girls, with a mean age of 12 years with a standard deviation of 2.6. The proportion of spectacle wear non-compliance was 19.5% (n=39). Children with non-compliance were more likely in the age group of 14-16 years (n=20{51.3%}; p=0.039). The main reasons for non-compliance were dislike to wear spectacles (28.2%), broken spectacles (23.1%), spectacles causing headache (20.5%), spectacles lost and parents' disapproval (20.5%), and peer pressure/teasing (15.4%). Significant difficulties faced while wearing spectacles were pressure on the nose due to worn-out nose pads (36.4%), pressure on ears causing pain in the temple and headache (34.1%), repeated cleaning of spectacles (29.5%), heavy spectacles (18.2%), excessive glare and pain in the eyes (12.5%), and improper fitting of spectacles (11.4%).

Conclusions

We found that non-compliance was more significant in school-going children aged 14-16 years and girls. The main reasons were unlikeness to wear, broken spectacles, headache, and spectacles lost. School children were not compliant because of many issues that should be addressed, and this information will be used for better eye care in school-going children with refractive errors.

## Introduction

People who have defective vision need spectacles to see properly [1]. Common indications for wearing spectacles include myopia, hypermetropia, astigmatism, and frequent headaches, which get worse on concentrating on a particular object, especially when reading, for certain distances when there is a doubling of objects or people, halos around lights, and need to squint sometimes [2].

Visual acuity (VA) is a measure of the ability of the eye to distinguish shapes and the details of objects at a given distance [3]. Vision impairment means that a person's eyesight cannot be corrected to a "normal" level. Vision impairment may be caused by a loss of VA, where the eye does not see objects as clearly as usual [4]. The International Classification of Diseases 11 (2018) classifies vision impairment into two groups: distance vision impairment (mild - VA worse than 6/12-6/18, moderate - VA worse than 6/18-6/60, severe - VA worse than 6/60-3/60, and blindness - VA worse than 3/60) and near-presenting vision impairment (near VA worse than N6 or M.08 at 40 cm) [5].

Uncorrected refractive errors are a significant cause of morbidity globally. Recent data show that uncorrected refractive error was among the leading causes of the global population's moderate or severe vision impairment in 2015 [6]. Wearing spectacles, in addition to being the most effective treatment modality for correcting refractive error, is also an economical treatment modality aiming to improve eye vision, function, and productivity in child subjects [7].

Various studies have assessed the factors governing compliance towards spectacles wear in children. It was seen that compliance is only seen in one-third of the subjects. Low compliance rates were seen even in old children and subjects with free spectacles. Factors responsible were self-esteem, safety concerns, peer pressure, perception of subjects and parents, forgetfulness, loss, breakage, and poor follow-up [7].

Globally, at least 2.2 billion people have near or distant vision impairment [5]. Almost 18.9 million children under 15 are visually impaired globally. In developing countries, 7%-31% of childhood blindness and visual impairment is avoidable, 10%-58% is treatable, and 3%-28% is preventable [8]. In Pakistan, the prevalence of children with vision impairment and refractive error was 5.4% and 5.3%, respectively [9]. In Rawalpindi District, Pakistan, 59% of school-going children aged 11-16 years were not compliant with full-time spectacle wear [10].

This study aims to determine the non-compliance of spectacle wear among school-going children and assess the factors responsible for this, which may be helpful to improve compliance and, ultimately, the population's quality of life and reduce the burden on the healthcare system.

## Materials and methods

The present cross-sectional study assessed the factors governing non-compliance to spectacle wear in children aged 5-16 years with refractive error history of more than six months. The study was conducted in different primary and secondary schools in Lahore, Pakistan, after obtaining ethical consent from the Institutional Review Board of Services Institute of Medical Sciences, Lahore.

The study included 200 children from both genders aged 5-16 years who had refractive errors and were prescribed to wear spectacles to correct refractive errors for more than six months. The study lasted three months, from July 2022 to September 2022. The inclusion criteria were school-going children aged 5-16 years with a refractive error history of more than six months, and the refractive errors included were myopia, hypermetropia, and astigmatism. The exclusion criteria were children with other causes of refractive errors not mentioned above, refractive errors secondary to systemic diseases, and children who wear contact lenses. The consent was obtained both from administrators of schools and parents of the children after a complete explanation of the study design and data collection tool.

For data collection, a detailed, structured, standardized questionnaire was used to collect the information from the students. All the respondents gave written informed consent, face-to-face interviews were conducted, and close-ended questions were asked. The questionnaire was translated into the local language (Urdu). The researchers themselves collected all the data. Collected data were subject to statistical analysis by Statistical Product and Service Solutions (SPSS, version 26; IBM SPSS Statistics for Windows, Armonk, NY). In analysis, for quantitative variables, mean and standard deviation were calculated. For qualitative variables, frequency and percentage distribution tables were generated. The chi-square test was applied to qualitative variables, and the p-value was calculated. A p-value of 0.05 or less was taken as significant.

## Results

A total of 200 children were included in the study. Demographic characteristics of the children are listed in Table 1. The study included 41 (21%) males and 158 (79%) females. There were 126 (63%) between five and 13 years and 74 (37%) between 14 and 16 years of age, with a mean age of 12 years, with a standard deviation of 2.6. A total of 174 (87%) had myopia, six (3%) had hypermetropia, and 20 (10%) had both refractive errors. Most of the children had refractive errors and had been prescribed spectacles for at least one year (21.5%), while the mean duration of refractive error and spectacle prescription was 3.4 years, with a standard deviation of 2.6.

**Table 1:** Demographic Characteristics of Respondents

Characteristics of Respondents			
		n	%
Gender	Male	42	21
	Female	158	79
Age Group	5-13 Years	126	63
	14-16 Years	74	37
Compliant	Yes	161	80.5
	No	39	19.5
Type of Refractive Error	Myopia	174	87
	Hypermetropia	6	3
	Both	20	10
Duration of Refractive Error (in Years)	0.5	21	10.5
	1	43	21.5
	1.5	2	1.0
	2	23	11.5
	3	25	12.5
	4	26	13.0
	5	19	9.5
	6	19	9.5
	7	6	3.0
	8	6	3.0
	9	2	1.0
	10	5	2.5
	11	1	.5
	12	2	1.0
Difficulty in Wearing Spectacles	Yes	88	44
	No	112	56

**Figure 1:**
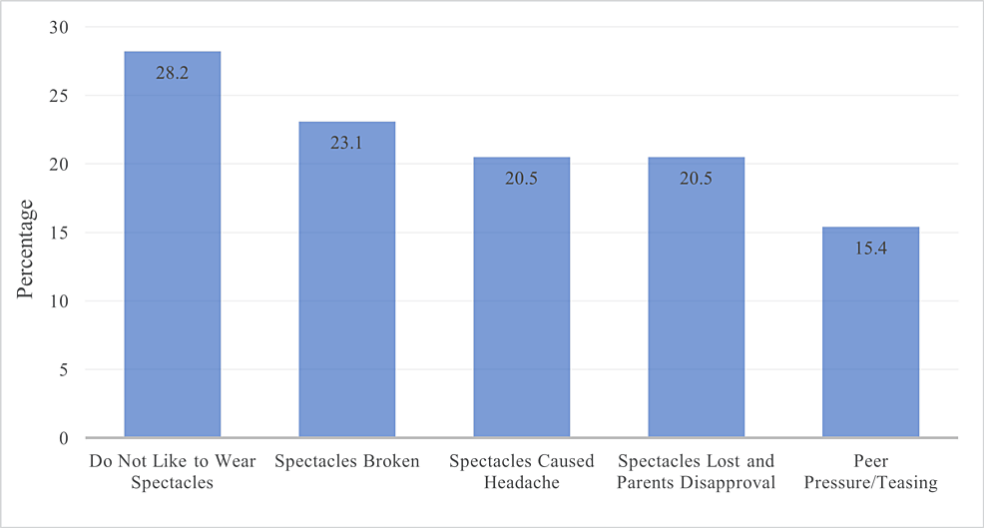
Causes of Non-compliance in Spectacle Wear

Overall, 161 (80.5%) children were compliant with spectacle wear, while 39 (19.5%) were non-compliant. The study results on assessing the reasons behind non-compliance are summarised in Figure 1. A dislike to wear spectacles was seen in 11 (28.2%) children. Broken spectacles were seen as the cause of non-compliance in nine (23.1%) children. Headache caused by spectacle use was responsible for not wearing spectacles in eight (20.5%) children. Spectacle loss and parents' disapproval were reasons for not wearing spectacles in eight (20.5%) children. Peer pressure/teasing was a cause for non-compliance in six (15.4%) children.

**Figure 2:**
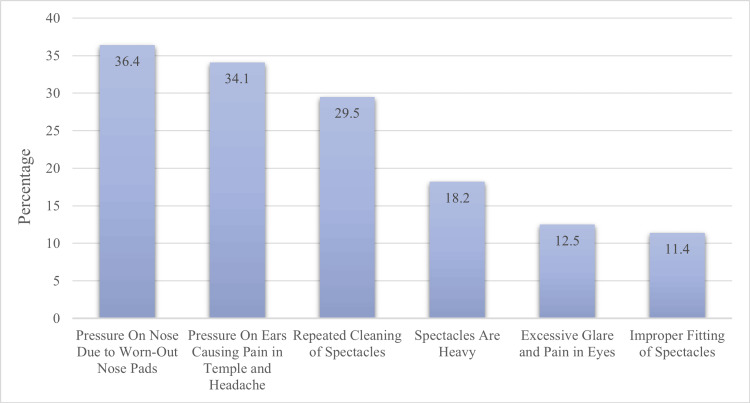
Causes of Difficulty in Wearing Spectacles

A total of 88 (44%) children reported difficulty in wearing spectacles, while 112 (56%) reported no problem with its causes (Figure 2). Worn-out nose pads causing pressure on the nose were reported by 32 (36.4%) children, and 30 (34.1%) children said pressure on the ears caused temple pain and headache. Repeated cleaning of spectacles caused difficulty for 26 (29.5%) children, and 16 (18.2%) children reported difficulty due to heavy spectacles. Excessive glare and eye pain caused difficulty in wearing spectacles for 11 (12.5%) children, and improper fitting of spectacles caused difficulty for 10 (11.4%) children.

The statistical analysis of the study is presented in Table 2. The children who were in the elder age group (14-16 years) were found to be more non-compliant (20, 51.3%) than those in younger age groups (5-13 years; 19, 48.7%). These differences were statistically significant (x2=4.239, p=0.039, df=1).

**Table 2:** Association of Demographic Factors and Non-compliance of Spectacle Wear

	Non-compliant (%)	Compliant (%)	Total (%)	P-value*
Age Group				
5-13	19 (15)	107 (85)	126 (20)	0.039
14-16	20 (27)	54 (73)	74 (37)	
*p-value <0.05; Chi-square test				

## Discussion

The present study aimed to determine non-compliance to spectacle wear in school-going children and its causes in schools in Lahore, Pakistan. The non-compliance was 19.5%, like a study conducted in Central India, in which the non-compliance was 20%, so the results of both studies were similar [11]. The non-compliance was less than in the study conducted in Rawalpindi District, Pakistan, in which the non-compliance was 59% [10], which could be attributed to an increase in awareness of the beneficial effects of the proper compliance of spectacles.

In our study, girls (89.7%) showed greater non-compliance than boys (10.3%), whereas in a study conducted in Qaasim Province, Saudi Arabia, boys (56%) were more non-compliant than girls (44%) [12]. Another study conducted in Uttar Pradesh, India, also showed greater non-compliance in boys (51.9%) than in girls (48.1%) [7], and these results may be attributed to the fact that these studies had a much-balanced distribution of gender as compared to ours. However, in the research conducted in Rawalpindi, Pakistan, the non-compliance was greater in girls (42%) than in boys (16%) [10].

The non-compliance was found to be greater in the older age group of 14-16 years (51.3%), which was like a study conducted in Concepción, Chile, in which the spectacle compliance decreased with increasing age, which least compliance in children older than 14 years of age (46%) [13]. However, in another study conducted by the World Bank in Pakistan, the compliance was more significant in the older age group of 14 years and above [9], which is also on par with the research conducted in Rawalpindi in 2017 in which the compliance was more significant in the older age group of 14-16 years than those in younger age groups [10]. This change in trend could be attributed to increasing insecurity about personal physical appearances in public among older children and an urge to appear more attractive and appealing on social media.

The major reasons for non-compliance in our study were dislike to wear spectacles (26.2%) and broken spectacles (21.4%), which was similar to a study conducted by the World Bank in Malawi, Nigeria, and Pakistan, in which the significant reasons reported for non-compliance were broken spectacles, dislike to wear spectacles, and parents disapproved of spectacle wear [9]. Similar reasons for non-compliance were reported by von-Bischhoffshausen et al. in a study conducted in Concepción, Chile, in which the main reasons for non-compliance were broken spectacles (22%) and dislike of wearing spectacles (21%) [13]. In a systemic review and meta-analysis of 23 studies consisting of 7,859 children conducted by Dhirar et al. in 2020, the primary reasons for non-compliance were personal factors, such as dislike for wearing spectacles (25.78%) and broken or lost spectacles (23.34%) [6], which are the same as reported in our study. This shows that major reasons for spectacle non-compliance remain the same worldwide, with a minor difference among the proportions of several reasons and causes.

The major strength of our study was that we obtained data from different schools all over the city. We also obtained data on difficulties while wearing spectacles, ultimately leading to non-compliance. Hence, we tried to fill this gap because no other study has addressed this issue, according to our knowledge. The limitation of this study was that the study was conducted exclusively in private schools in urban settings affecting the generalizability of the results in lower and middle-income countries (LMICs).

## Conclusions

The non-compliance among school-going children is because of a dislike of wearing spectacles and broken spectacles. To improve compliance in school-going children, the difficulties faced during spectacle wear should be addressed, such as the pressure on the nose due to worn-out nose pads, pressure on the ears causing pain in the temple, and repeated cleaning of glasses/spectacles. The involvement of parents and other caregivers in addressing these issues will improve eye care and compliance with spectacle wear in school-going children with refractive errors.
